# Clinical Notes

**Published:** 1871-08

**Authors:** F. K. Bailey

**Affiliations:** Knoxville, Tenn.


### Clinical Reports.

### CLINICAL NOTES.

By F. K. BAILEY, M.D., Knoxville, Tenn.

Case I.—Rheumatism, with Dermatoid Complications.—Was called, *May 7th, 1871*, to see a man, about 40; sanguine nervous temperament; occupation, photographer; laboring under a painful affection of the muscles upon the right side of the neck. There was heat, redness, and swelling, extending from the top of the shoulder to the mastoid region, and reaching to the sternal extremity of the clavicle. On moving the head the pain was much aggravated, and most obvious along the course of the sterno-cleido-mastoideus muscle. In addition, there was a tumefied condition of the integuments of the right frontal region, stopping abruptly at the mesial line, and extending upwards beyond the line of the hair. The skin upon the forehead was red, and of a shining appearance. Just above the right eye was seen a spot about eight lines in diameter, circular in form, and elevated above the surrounding parts, already swollen, as stated above. Upon the surface of this circular spot were minute vesicles, and presenting all the characteristics of herpes phlyctænodes. There was also general febrile excitement, with heat of surface, pulse increased in frequency, constipation, and scanty red urine. There was shooting pain in the neck, and severe headache, worst on the right side.

I prescribed a saline cathartic, following up with a mixture as follows:

℞. Iod. Potassii------------------------------ʒij.

Syr. Sarsap. Comp.---------------------℥ij.

Tinc. Gentianæ--------------------------℥ss.

M. Sig. Teaspoonful three times daily. To bathe the forehead and neck with a solution of hyposulphite soda.

There was but little abatement of symptoms for four days, but on the 11th, the pain and swelling had nearly left the neck, and the herpetic spot had scaled over, leaving the surface rough. The integuments of the forehead were still swollen and painful, and the headache continued severe.

It was about ten days from the first attack before the affected parts had resumed their normal appearance.

The general symptoms in this case closely resembled those in herpes zoster, besides the local manifestations were confined to one side. I will add that this gentleman has for many years labored under a hypertrophy of the heart, but without marked valvular lesion.

Case II.—*April 10th, 1871, 8 P.M.* Called to visit a light mulatto woman, aged about 32; married, but never has been pregnant. Complaining of severe sore throat; the pharyngeal mucous membrane much congested, and both tonsils enlarged; little heat of surface; pulse 80, and soft; pain in the lumbar region, with dysuria; bowels open. Prescribed:

℞. Spts. Nit. Dulc. ---------------------------ʒj.

Tinc. Belladonna---------------------------ʒj.

M. Sig. Teaspoonful every three hours. To steam the throat.

*11th, 7 A.M.* Patient states that the soreness of the throat was relieved after taking the first dose of the mixture, but feels this morning a prickly sensation in the fauces, extending downwards; urine more free. It was more than a week before she was able to go about much, but before the end of the month was nearly well. On the 1st of May she was taken with rheumatic pains in the chest, and extending around and through the epigastric region, aggravated on sneezing, yawning, or making any movement which involved the action of the diaphragm. Pain also along the course of the œsophagus, aggravated by the act of swallowing; there was heat, pain, and swelling of the wrists, knees, and ankles; pulse accelerated, with palpitation and difficulty of breathing when lying down; bowels constipated, and urine very red and scanty.

This condition continued until the 8th, unchanged, when there appeared an eruption of urticaria upon the limbs and body, attended with a deep shooting pain, extending "to the bone," wherever a spot appeared.

On the 10th, found her complaining of severe pain in the cardiac region, attended with increased impulse. Learned that she had for years been troubled with palpitation, particularly during the menstrual period, and a victim of dysmenorrhœa from puberty. Had taken for some days a mixture of spts. nit. dulc. and tinc. opii. camph., but to-day prescribed:

℞. Iod. Potassii--------------------------------ʒij.

Bicarb. Potassa---------------------------ʒiij.

Aqua-----------------------------------℥ij.

Spts. Etheris Comp.-------------------ʒij.

M. Teaspoonful every four hours. Sul. quinine, in 2-gr. doses, alternated; applied belladonna plaster to the cardiac region; two comp. rheu. pills at night.

*11th.* Pain somewhat relieved, but cannot tolerate quinine, as it causes singing in the ear. Was obliged to remove the belladonna plaster, as it caused faintness, which is not an uncommon occurrence in some persons; complains of an unpleasant sensation in the whole pharyngeal region, which may arise from the action of belladonna.

To continue the iod. potass mixture, and, to cause sleep, at 3, 6, and 9 P.M. a teaspoonful of the following mixture:

℞. Brom. Potassii ........................ ʒij.

Aqua Puræ ........................ ℥j.

Spts. Etheris Comp. ........................ } āā ℥ss.

Fluid Ext. Valerian ........................ }

An enema at bedtime of warm water, with a tablespoonful of a mixture containing equal parts of tinc. asafœtida and valerian.

*12th.* Some better; rested well; less pain and uneasiness in the cardiac region; eruption not so obvious; urine more free; some pain in the sacral region. To continue the iod. potassii and bromide mixtures, and take oyster soup.

*14th.* Able to sit up, but has felt for two days much of the pain and distress in the region of the diaphragm, which is aggravated by all the movements wherein that muscle is called into action. Urine still rather scanty. Sweating very freely at night. To take a 2-grain quinine pill every four hours, and to continue the mixtures.

*16th.* Much improved, except that the urine is still scanty and very red, with an acid reaction. Has rested very well for some nights past. Pain nearly subsided in the epigastrium, but continues in the lumbar and iliac regions.

*17th.* Much better; urine very free, which may be a result of taking some gin yesterday and last night.

*20th.* Has improved since last report, but complains of pains in the feet and hands, which are somewhat bloated; has had no pain in the chest or region of the diaphragm for some days; urine is passing off freely. The bowels have been kept open for the last week by means of enemata. Has taken the iod. potassii mixture regularly, but the bromide was suspended after the 16th.

*27th.* Gradual improvement during the past week. There has been more or less pain in the joints of the extremities. The action of the kidneys has been variable, and as the rheumatic pains have been less when the urine was secreted the most freely, the conclusion is that the primary fault was in the renal function. The patient has been subject to rheumatism since puberty. In *ante bellum* days a slave, she was, when work crowded, sent into the field with others more muscular. A slender yellow girl can endure but little exposure and physical exertion, and this individual soon contracted rheumatism. Cardiac complications obtained, and dysmenorrhœa, mitral hypertrophy, mucous congestion, leucorrhœa (both uterine and vaginal), and prolapsus, made up a catalogue of ills, which will be a source of anxiety and suffering in all her future life.

In detailing rather cursorily the pathological condition of this patient, the history of hundreds is told, that contribute to the clinical practice of this and all other Southern localities, so far as I have been able to observe and learn. This state of things is not confined to the colored population. We see great numbers of white women who are similarly affected.

In the *Half-yearly Compendium of Medical Sciences*, for Jan., 1871, is an article by Dr. S. Baruch, of Charleston, S. C., from the *Richmond and Louisville Medical Journal*, for December, 1870, in which is expressed my own views upon the causes which bring so much suffering upon the lower classes of women in the South.

He says (1), "Ignorance of the laws of nature, whose infringement is so often followed by deplorable consequences." In this connection he alludes to the common practice of leaving the bed too soon after confinement, and before the uterus has even began to return to its former tone and condition. This is an obvious fact in all localities among the poorer classes, who are compelled to leave the lying-in bed in order to give attention to household affairs. It is particularly so in warm climates, where the general system is relaxed, and the process of involution is tardy. Although the heat is less in this region than in the Gulf States, still uterine disease is fearfully common. One very common cause of disease follows from early childbearing. A great many of the women among the lower classes became pregnant as soon as ovulation occurred. This is true not only among the blacks, but the whites. It is very common, when inquiry is made, how old was you when your first child was born, for them to say "thirteen or fourteen."

Exposure to cold and dampness is a fruitful cause of rheumatism among all ages and both sexes. Especially do we find it in young girls at the age of eight, and cardiac complications are not uncommon before puberty. Rheumatic dysmenorrhœa in such subjects will occur upon the first appearance of the menses. Amenorrhœa is frequent, and upon each recurrence of any disturbance of the menstrual function, there is local congestion, which in a short time causes hypertrophy of the uterine walls, and a catarrh of the mucous lining.

Not long ago a girl of fifteen, from the poor white class, came to my office complaining of excessive pruritus of the vulva. On inquiring, found she had leucorrhœa, and she told me she had had it since her first remembrance. She seemed to regard that as a normal condition.

Case III.—Uterine Tumor.—Was called, *March 6th, 1871*, to visit Mrs. S——, a woman of 45, mother of six children, youngest four years old. Found her looking pale and dejected, and complaining of severe pain in the pelvic region. In the hypogastrium could be felt a hard tumor, but not painful to the touch. On vaginal examination, the os uteri was found low down, thickened, and immovable. Could be felt within the os, which was patulous, a firm tumor as large as a child's head at full term. The uterus was firmly fixed—complete immobility. The tumor could be felt about 18 lines from the os. No pressure at all upon the bladder. The urinary organ not disturbed, either mechanically or functionally. A grumous watery discharge from the vagina. On introducing the finger to the rectum, found that organ crowded by the tumor; could not move it in the least by firm pressure of the finger. Learned the following history, as related by the patient: In September last, the menses ceased, and nothing was seen till January 1st. At that time the bloody flow above-mentioned commenced, and has not ceased at all, and sometimes lumps the size of grapes would pass. The enlargement was not noticed by her till the middle of December. Having no speculum with me, merely advised the free use of warm water injections to move the bowels, which, as will be inferred, had been evacuated very irregularly.

I will here mention that during the last six weeks there had been great difficulty in defecation, with severe vomiting, which, from her account, might have been stercoraceous.

*7th.* Bowels had moved freely after the enema was used. Patient says great relief was experienced from a feeling of distension through the whole abdominal region. From her further relation of former symptoms, I became satisfied that at times there had been a mechanical obstruction of the rectum by the pressure of the tumor, causing the same train of symptoms as found in strangulated hernia. Further, says that the urine has generally passed away very copiously, compelling her to void it every hour through the night.

Introduced a speculum, and found the cervix much thickened. The os was patulous, and presented a pouting appearance. Introduced a sound, which encountered the tumor at a distance of less than two inches. It had a firm inelastic resistance to pressure with the sound, and attended with no pain. The flow so free as to fill the speculum, and requiring frequent mopping out with cotton. On inspecting the cervix closely, found that it was indurated, and the mucous membrane livid and very much congested. Applied solid nitrate of silver freely to the cervix, and advised frequent injections per vaginum of warm water, and to keep the bowels open by means of enemata.

*9th.* Condition unchanged since last report, except that the strength is failing. The pain in the sacral region constant and unabated. No relief from laudanum injections per ano on my visit yesterday. Bowels moved with difficulty. The tumor appears to almost completely obstruct the rectum. Vaginal discharge still very copious.

*11th.* No improvement. The cervix less indurated, but appearance of sloughing from the os. Introduced a sponge tent upon the right side, and anteriorly somewhat to the tumor, after cauterizing the mucous membrane freely. The bowels move more freely for two or three days. Prescribed anodyne enemeta to vagina, with dilute carbolic acid added to correct fœtor. To give milk punch, and beef soup.

*12th.* Removed sponge tent. Os more dilated. Passed a sound three inches to the right and anteriorly to the tumor. Sloughs of the size of a nutmeg coming away. Touched freely with nitrate of silver, and crowded up a pledget of cotton wet with sol. carbolic acid. There is less fœtor to-day, and the discharge was lighter colored which followed the removal of the sponge tent. Patient very feeble; pulse small, but frequent.

*13th, 12 A.M.* Very feeble; just recovering from a fainting turn. Removed pledget of cotton, which had remained unmoved. Gave stimulants and tonics, and enjoined absolute rest.

*14th.* Patient died at 11 P.M. last night. She had not moved her lower limbs after fainting yesterday. In this case I had given an unfavorable prognosis from the first, and examined her more for purposes of diagnosis than an expectation of affording relief.

Up to my being called no medical aid had been sought. The woman had taken large quantities of tinc. opii on her own responsibility to relieve pain, with the effect to render the bowels torpid, and add to the trouble. I could detect no signs of peritonitis or metritis, and death undoubtedly resulted from exhaustion. If timely aid could have been rendered, the tumor might possibly have been reduced by enucleation, as it was so near the os as to be easily accessible. It had evidently been rapid in its formation and growth, or existed for a period without being noticed.

Case IV.—Epithelioma.—*March 11th, 1871.* While in attendance upon the last-mentioned patient, I was requested to see Mrs. B——, aged 47, mother of several children. Has labored under disease of the uterus for five years. States that in 1867 there was ulceration of the neck and mouth of the womb. Was treated by a physician in the city, and she understood that the neck sloughed away. On attempting a vaginal examination, found the finger could not be passed more than an inch and a-half, as it encountered cauliflower excrescences. The parts extremely tender to the touch, and bleeding freely. There has been this state of things for some months. Did not think it advisable to attempt the introduction of a speculum. The woman has for so long a time suffered severe pain in the pelvic region, together with constipation, and irritable stomach, that she is emaciated and exhausted from want of nutrition, as well as loss of blood. Did not consent to treat the case, for the reason that from want of means, as well as appreciative intelligence, the carrying out of any definite plan could not be relied upon. Advised the injection per vaginum of a weak solution of carbolic acid, to correct fœtor, and the use of tonics and nutritious food.

Another physician was subsequently called in, who soon came to the same conclusion with myself, and I learned that she was slowly sinking at latest accounts (June 10th).

Case V.—*March 6th, 1871.* Mrs. B——, aged 43, fleshy, and looking tolerably well, mother of nine children, youngest nine years old, has been irregular since May, 1870. Menses scanty when present at all, with leucorrhœa most of the time. Pain in the sacral region nearly constant. Having some "show," deferred further investigation of the case for the present.

*15th.* Called, and was told that she was pronounced pregnant by a physician in June last. There was abdominal enlargement, swollen mammæ, and other signs. There was some milk in the breasts after November till February, with increase of size till some time in January. Since that time she has noticed a lessening of the abdominal fulness, with flow more or less colored at times till the present.

On palpation externally, found fulness in the right side as high as the umbilicus. Vaginal touch found the os uteri lying in the hollow of the sacrum. Introducing a speculum, found the os looking backwards, cervix thickened, pale and turgescent. Os patulous, with a small quantity of bloody mucus passing out. Introduced a sound about four inches, anteriorly and to the right side, without encountering any obstacle.

From the history of the case, with present appearances, it may have been one of blighted fœtus—spurious pregnancy. Involution taking place slowly. Touched the cervix with nit. silver, and prescribed as follows:

℞. Brom. Potass ℥ss.

Aqua Puræ ℥j.

Spts. Etheris Comp., Fluid Ext. Ergot, āā ʒij.

Tinc. Cinch. Comp. ℥iss.

M. Sig. Half a teaspoonful morning and night. To exercise moderately, and keep the bowels open with enemata.

*May 1st.* Since last report the woman has been improving in strength, with lessening of size.

*June 1st.* Entirely recovered. There has been complete restoration of uterine, health, and function.

Case VI.—*June 7th.* Was invited by Dr. Boynton to call with him upon a woman about 45, nervous, sanguine. Had her last child about twelve years ago. Had been in tolerable health till within a month, when she began to feel a severe pain in the pelvic region, extending down the right thigh. A few days ago, the distress became so severe as to induce spasm, upon which medical aid was sought.

On palpation, found a hardened fulness in the hypogastrium, somewhat uniform in shape. On vaginal examination, the cervix was hard, somewhat enlarged, and the os very small, and feeling firm, like a button-hole in a vest made of thick, firm cloth. On speculum inspection, found the cervix congested, livid, and about two inches in size. Slight discharges of a grumous appearance. Passed a sound anteriorly and to the right side of the uterus, about two inches and a-half, when a firm inelastic body was encountered. Passing the finger into the rectum, found the enlargement to occupy the hollow of the sacrum. With the exception of the constriction of the os, found the case very similar to Case III. The tumor doubtless fibroid, and of rapid formation.

No measures adopted except by way of palliation in this case. Full doses of morphia will give relief for some hours.

Case VII.—Syphiliphobia.—This is an affection occasionally mentioned by writers, but, perhaps, not of frequent occurrence.

As the term implies, there is an apprehension on the part of the patient that syphilis is present in some form or other, without any clear evidence of its existence, but a consciousness of having been exposed to its contraction, or a fear that some seeds of a former manifestation still remain in the system.

In *October*, *1867*, a young man came to my office, in company with a friend. He presented a downcast appearance, restless, with a quick and rather frequent pulse. He at once began to tell me that he had syphilis, and that he knew it would kill him. I examined him very carefully, with a view to detect any physical signs, but could see none. There were some cicatrices upon the genitals, which were the remains of chancres, but the healing was complete. There was no soreness in the throat, nor nodulated eminences upon the limbs. He complained of a confused pain in the head, and an unpleasant sensation in the back of the neck. When asked if he had pain in the shins on lying down at night, he replied in the affirmative.

From his attendant, I learned that in 1865 he contracted syphilis, and was treated promptly, and, as it was then supposed, successfully. All traces of primary symptoms had long ago disappeared.

During the summer of 1867, he began to express apprehensions that he still was laboring under the effects of the disease, and soon became, as his family thought, insane upon the subject. When I first saw him he evidently was in a morbid mental condition. His great fear was, that he was in an incurable condition; that it would break out anew, and that he must surely die. It was useless to attempt the allaying of his fears, no reasoning would pacify him. I gave him iodide potassii in small doses, with some bitter tonic, with full doses of Dover powder at night.

As he lived some twelve miles in the country, I did not see him for nearly a week. He then came in company with his father, who informed me that his mental condition was unchanged from the time of my first interview with him. The third visit was made about three weeks from the first, and when I last saw him his friends were taking measures for his admission to the insane asylum. Since that time I have heard nothing from him.

In the summer of 1869, I saw, at different times, an Irish woman of 40, or more, who was complaining of some uterine difficulty, together with a derangement of the liver and kidneys. In connection with the ailments enumerated, she had either in reality or imagination contracted syphilis some years previous. For some months during the summer she was constantly troubled in mind, for fear that some remains of the poison still lingered. It was the burden of her thoughts and fears, although there were no appreciable indications of such a condition.

In the summer of 1870, a large, fleshy, and vigorous girl of 24, came to my office to consult me in regard to her condition. I learned on inquiry that she had, like many others, been a victim to some man's lust, and, as she supposed, "got the bad disorder." I could not discover that she had any signs of syphilis at that time, or that she had ever been affected.

There was nothing indicative of any departure from a normal condition, being in as good health as any one could be. I prescribed, however, iodide potassii in small doses, with laxatives, which she took faithfully for some weeks before the idea was effaced from her mind that she was diseased.

Excepting the three cases above related, I have never known a person that was really diseased who manifested any great degree of solicitude as to the "situation." On the other hand, there is, perhaps, a want of due appreciation as to the present or future of the case. The general impression is that venereal diseases can be cured as readily as any other. Generally, the victims are persons more or less void of any scruple as to the *morale* of the conduct which induces venereal disease, and in very many, the chief anxiety about a removal of their trouble is to be able to plunge anew into vice. To see a person so affected as to be mentally deranged, or to imagine a disease like syphilis to be present, when there are no palpable signs of it, is not common, so far as my own experience has extended.

Since writing the above, I have conversed with one of our city physicians in regard to the first case, who says that the young man came to him some months before I saw him, and that he was then laboring under the same impression, but that there were no signs of syphilis, that he could discover.

The cases above related are thus presented, with no comments or opinions expressed, except what are involved in giving the facts. The like may have occurred in the practice of others, and, if so, it is hoped they may be reported.

